# Force vs. impulse: assessing the applicability of dynamic strength indices in individualized training recommendations

**DOI:** 10.5114/biolsport.2026.157994

**Published:** 2026-01-02

**Authors:** Yuhang Liu, Chris Bishop, Lachlan P. James, Changda Lu, Fanhao Meng, Xiaoyi Yuan

**Affiliations:** 1 School of Education, Beijing Sport University, Beijing, China; 2 London Sports Institute, Middlesex University, London, United Kingdom; 3 Sport, Performance, and Nutrition Research Group, School of Allied Health, Human Services, & Sport, La Trobe University, Melbourne, VIC, Australia; 4 School of Sport Science, Beijing Sport University, Beijing, China; 5 School of Strength and Conditioning, Beijing Sport University, Beijing, China; 6 The Key Laboratory of Sports Training, General Administration of Sport of China, Beijing, China

**Keywords:** Countermovement jump, Force platform, Resistance training, Strength diagnosis, Strength testing

## Abstract

In this cross-sectional investigation, we aimed to compare impulse-derived (iDSI) and forcederived (fDSI) dynamic strength indices to determine their consistency in guiding individualized training recommendations. It also assessed the agreement between training prescriptions based on these indices and analyzed individualized neuromuscular profiles through case study comparisons. Twenty male skeleton and bobsled athletes performed countermovement jump (CMJ) and isometric mid-thigh pull (IMTP) assessments in a counterbalanced order. Wilcoxon signed-rank tests were conducted to examine differences between fDSI and iDSI. Spearman correlation coefficients quantified intra-group relationships, and linear regression analysis evaluated the model fit between indices. Cohen’s kappa (κ) was applied to assess agreement in training classifications derived from fDSI and iDSI. Both indices showed limitations in representing lower-limb neuromuscular function (Z = –3.72, p < 0.01, large effect). A moderate correlation was observed between iDSI and fDSI (rs = 0.47, p < 0.05), with moderate agreement in their training recommendations (κ = 0.52, 95% CI: [0.38, 0.66]). Case-study analyses revealed substantial inter-athlete variability in CMJ force–time characteristics, highlighting the need for individualized interpretation within performance profiling. Incorporating phase-specific, multi-metric evaluations of force–time variables may improve the precision of training decisions and better inform athletespecific programming.

## INTRODUCTION

Assessing an athlete’s maximal force output and training adaptations is a central objective in sports science and strength and conditioning [1]. The Dynamic Strength Index (DSI) has emerged as a widely used metric for monitoring these adaptations [2], defined as the ratio between peak force outputs during dynamic and isometric tasks. Various methods exist for calculating DSI, including combinations such as the countermovement jump (CMJ) with the isometric midthigh pull (IMTP) or the squat jump (SJ) with the isometric squat (ISO) [3]. Among these, the CMJ–IMTP pairing is generally regarded as more reliable, as both tests exhibit high reliability, are technically easier to standardize, and together capture complementary aspects of maximal isometric force and dynamic explosive performance [4, 5].

Force-based DSI (fDSI), typically calculated as the ratio of CMJ peak force to IMTP peak force, aims to quantify an athlete’s ability to transfer maximal strength into dynamic movements [6]. A higher fDSI suggests better transfer of isometric strength to ballistic tasks, while a lower value may indicate strength dominance with limited explosive translation [5, 6]. However, fDSI’s reliance on peak force neglects the time-dependent characteristics of force production, such as duration and rate of force application [7].

Traditional CMJ metrics such as peak power or jump height provide valuable outcome-based information but do not sufficiently capture the neuromuscular strategies or time-dependent force characteristics underlying performance [8]. Therefore, additional metrics such as braking duration, propulsive impulse, and force at zero velocity are essential for identifying individualized movement strategies [9]. Alternative metrics—such as phase-specific durations (e.g., braking and propulsion), force at zero velocity, and power-time curve—provide richer insight into force application and fatigue management. These variables help characterize movement strategies beyond simple output values and are crucial for individualized training planning [10].

To enhance accuracy, strength assessment models should incorporate both peak force outputs and time-dependent measures, which reflect an athlete’s ability to generate and sustain force over a given duration [11]. Unlike fDSI, which relies on peak force values, iDSI (impulse-based DSI) is calculated as the ratio of dynamic impulse to isometric impulse, providing a time-sensitive representation of force production, as it reflects the accumulation of force over the propulsion phase [12]. Despite growing interest [13–15], few studies have systematically compared fDSI and iDSI in terms of their relationships to force-time variables or their consistency in generating training recommendations. Whether these indices lead to comparable athlete profiling or diverge in their implications remains unclear.

Therefore, the purpose of this study was to: (a) examine the relationships between fDSI, iDSI, and key force-time performance variables (e.g., force at zero velocity); (b) evaluate the consistency of training recommendations derived from both indices; and(c) provide case study comparisons of two athletes’ CMJ force-velocity profiles to visualize differences in jump strategies and force application. In addition to these aims, we sought to deepen the understanding of the biomechanical mechanisms underlying jump height, thereby supporting more precise and contextually informed athlete monitoring. By clarifying how force–time characteristics contribute to jump performance, this study aims to enhance the accuracy and specificity of performance evaluation and subsequent training decision-making.

## MATERIALS AND METHODS

### Study Design

This study employed a cross-sectional observational design to investigate the relationships between different DSIs and force–time curve performance variables, and to assess the consistency of training prescriptions derived from iDSI and fDSI. To minimize potential order effects, a counterbalanced testing protocol was implemented [16]. Testing sessions were conducted at the same time of day for each subject and performed under similar environmental conditions (temperature: ~21°C, humidity: ~60%).

To explore how differing DSI profiles may guide individualized training recommendations, a case study analysis was also conducted. Two athletes were selected based on the magnitude of discrepancy between their iDSI and fDSI scores—one with the largest difference, and one with the highest agreement. Their CMJ force-time curves were time-normalized and analyzed alongside key individualized metrics such as braking impulse, propulsive phase duration, and force at zero velocity. This approach allowed for a detailed examination of neuromuscular strategy differences and potential training implications.

### Participants

A total of 20 male bobsleigh and skeleton athletes voluntarily participated in this study. All participants were full-time nationalteam athletes competing at the highest domestic level, with regular participation in International Bobsleigh and Skeleton Federation (IBSF)–sanctioned events; two athletes had competed in both World Cup and Olympic competitions. All athletes had between 4 and 7 years of structured training experience (mean: 5.2 ± 1.1 years) and completed 8–10 programmed training sessions per week as part of their national team program. Demographic characteristics (mean ± SD) were as follows: age = 23.3 ± 1.7 years (21–26 years); body mass = 78.4 ± 6.7 kg (67.4–95.3 kg); body height = 1.83 ± 0.04 m (1.81–1.87 m); free-weight back squat 1RM = 145.9 ± 18.8 kg (110–175 kg); relative back squat 1RM = 1.86 ± 0.19 (1.58–2.17).

Athletes underwent a single testing session as part of an ongoing long-term monitoring program. All participants were free of musculoskeletal injuries or physical limitations that could affect performance. Prior to data collection, athletes completed three familiarization sessions, each separated by 72 hours, to minimize learning effects. Data collection commenced 72 hours after the final familiarization session. All participants were informed of the study procedures and provided written informed consent before participation. This study was approved by the Ethics Committee of Beijing Sport University (Beijing Sport University Ethics Committee of Sport Science) and conducted in accordance with the Declaration of Helsinki (IRB Approval: 2025093H).

Due to the elite nature of the participant group, recruitment opportunities were limited. Nevertheless, the sample size is consistent with applied sport science research where similar populations are typically investigated [17]. Thus, while acknowledging the limitations associated with small samples, the current design is justified for the study’s aims—namely, to explore variable relationships and the training relevance of different DSI in a performance-monitored elite cohort.

### Procedures

#### Experimental Testing Sessions

To minimize order effects, the CMJ and IMTP tests were counterbalanced across participants. Testing occurred during the early preseason phase over multiple sessions. Athletes completed a standardized dynamic warm-up: 20 minutes of treadmill running at 6 km/h (Quanzhou Shuhua Sports, China), followed by dynamic stretches (three sets each of thoracic rotation lunges and lateral lunges), and three maximal effort CMJs to activate neuromuscular function. All CMJ and IMTP data (excluding body mass and height) were used in the analysis. For the CMJ, participants received instructions and a familiarization trial. Athletes were cued to “jump as high and fast as possible with a quick, explosive countermovement” and performed all jumps with hands on hips to eliminate arm swing. Countermovement depth was self-selected but performed rapidly to a comfortable range. Up to five trials were completed; invalid trials (e.g., arm swing, delayed descent, or inconsistent foot positioning) were discarded and repeated. Foot placement was standardized using tape markers.

For the IMTP, participants received technical instructions and one familiarization trial. Athletes then performed two submaximal pulls (at ~50% and ~75% perceived effort), with one-minute rest intervals between attempts. Testing was conducted on a custom IMTP rack, with hip and knee angles set to ~140°–150° and ~125°–145°, respectively, confirmed with a handheld goniometer. Foot placement was self-selected but standardized across trials. Lifting straps secured grip to minimize grip strength influence. Trials began with a countdown and a verbal cue to “break the bar upward” while driving through the floor, as described by Comfort et al. [18]. Athletes maintained posture and joint angles, with maximal force sustained for ~3–4 seconds. Verbal encouragement was provided during each trial, and one-minute rest intervals were enforced.

#### Measurement Equipment and Data Analysis

Vertical ground reaction force (vGRF) data were collected at 1,000 Hz using a force plate system (Kistler Instrument Corp., Shanghai, China) [19]. For CMJ trials, GRF signals were summed into a single vector [20]. IMTP data were similarly recorded at 1,000 Hz using the same force plate. The average of valid trials was used for iDSI and fDSI calculations to reduce the impact of performance variability and typical error [21]. Bioware 5.11 software (Kistler Instrument Corp., Shanghai, China) was used to process the data. Participants were instructed to stand still during the first second of the data collection period to determine their body weight. The raw vertical forcetime data from each jump trial were exported as text files and analyzed using a custom Microsoft Excel spreadsheet (2024 edition, Microsoft Corp., Redmond, WA, USA) [22].

**FIG. 1:**
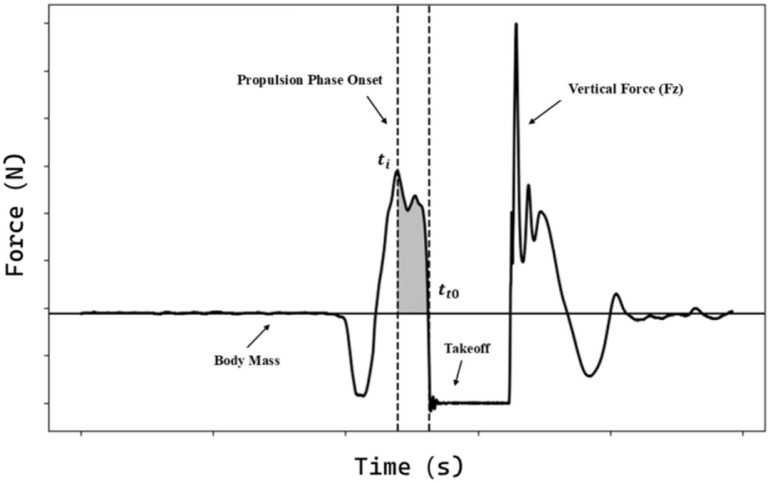
Visual representation of the CMJ impulse calculation area.

Impulse, defined as the integral of force over time, represents the area under the force-time curve. It quantifies the total force output during a specific phase of movement. Here, t_i_ represents the onset of the propulsion phase, and t_t0_ marks the takeoff moment (Figure 1). The impulse equation is given as:


$$Impulse=\int_{t_{i}}^{t_{t0}}F_{Net}dt$$ (1)

where F_Net_ is the net force, defined as the difference between ground reaction force (F_GRF_) and body mass force (F_BM_):


$$F_{Net}=F_{GRF}-F_{BM}$$ (2)

Velocity at the start of movement was calculated using the standard kinematic equation, where is initial velocity, is acceleration, and is time. During the transition from the braking to propulsion phase, vertical velocity momentarily reaches zero. This point marks the reversal of movement direction and is defined as the onset of the propulsion phase [23]. Velocity (V) was derived from the impulse equation:


$$V=V_{0}+at$$ (3)

Key movement phases of the CMJ were identified using synchronized force-time and center-of-mass velocity data, based on the criteria described by McMahon et al [23]. The braking phase was defined as the interval from the initiation of downward movement—identified when vGRF deviated by more than ± 5 N from body weight—to the lowest center-of-mass position, where vertical velocity equals zero. The propulsion phase was defined as beginning at this lowest point and ending when vertical force fell below body weight, signifying toe-off and the cessation of net positive force production. These operational definitions enabled consistent phase segmentation for the calculation of impulse and associated forcetime variables across all participants.

The impulse integration period was defined based on the onset of the propulsion phase t_i_ and takeoff t_t0_:

$${\text{Impulse}}_{CMJ}=\int_{t_{i}}^{t_{t0}}F_{GRF}\;dt-\int_{t_{i}}^{t_{t0}}F_{BM}\;dt$$ (4)

**FIG. 2:**
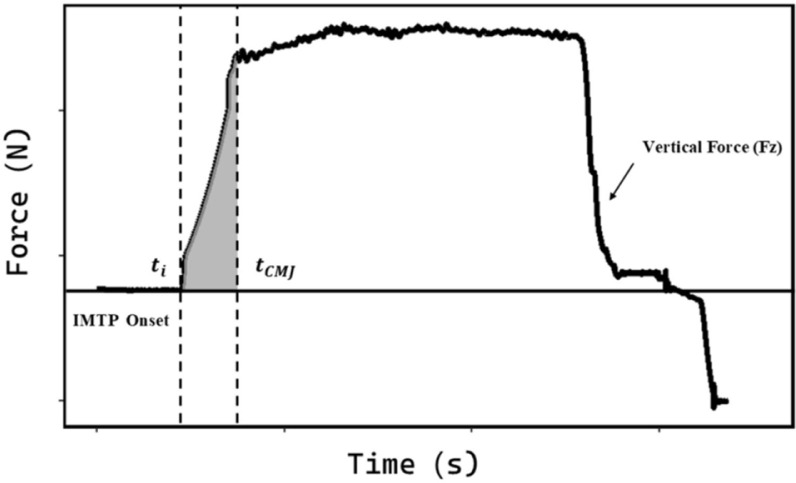
Visual representation of the IMTP impulse calculation area.

Impulse during the propulsion phase of the CMJ and during a matched-duration isometric force application window of the IMTP was calculated as the area under the net force-time curve (i.e., force minus body weight), using the trapezoidal method [24]. To ensure individualized comparisons, the IMTP impulse integration window was adjusted for each subject to match their CMJ propulsion impulse duration (Figure 2) [25]. The equation used was:math

$${\text{Impulse}}_{IMTP}=\int_{t_{i}}^{t_{CMJ}}F_{GRF}\;dt-\int_{t_{i}}^{t_{CMJ}}F_{onset}\;dt$$ (5)

where t_i_ and t_CMJ_ represent the onset and the corresponding impulse duration of the CMJ test, respectively.

IMTP trials were excluded from analysis if they lacked clear maximal intent, as indicated by delayed force development or abnormally low peak force values.

#### Study Analysis

Correlation analyses were conducted between iDSI, fDSI ratios, and force-time performance metrics, including: F_V0_: Force at zero center-of-mass velocity (N); BD: Braking phase duration (s); PD: Propulsion phase duration (s);TD: Total duration (s); MBP: Mean braking phase power over time (W · kg^−1^ · s^−1^); MPP: Mean propulsion phase power over time (W · kg^−1^ · s^−1^); II: Impulse from IMTP (N · s^−1^); IPF: IMTP peak force (N); PPF: CMJ Propulsion phase peak force (N). Linear regression models were applied to examine the relationship between iDSI and fDSI and determine the strength of their predictive equations. Given the number of correlation analyses performed, we applied a family-wise error correction to control Type I error. The Bonferroni correction was used, adjusting the significance threshold to α = 0.05/9 = 0.0055. Correlations were considered statistically significant only if the adjusted p-value fell below this threshold. Alternatively, results were also reviewed under the Benjamini-Hochberg procedure to balance false discovery risk.

To evaluate the consistency between fDSI and iDSI, athletes were classified according to their DSI values. Specifically, athletes with a DSI value greater than 0.80 were categorized as requiring a maximal strength–focused prescription, whereas those with values below 0.60 were categorized as requiring a ballistic/dynamic force–focused prescription [26]. Agreement was defined when both indices assigned an athlete to the same training category, while disagreement was defined when the indices produced different classifications. Case study analyses were conducted on two athletes with the closest and greatest differences in iDSI and fDSI values, focusing on their specific force-time profiles. A percentile ranking scale (Table 1) was constructed to further examine force-time characteristics. Additionally, time-normalized CMJ force-time curves were included for a visual comparison of jump characteristics and strategies.

**TABLE 1:** Descriptive statistics and percentile distributions for the CMJ and IMTP test variables, along with their respective dynamic strength indices (DSIs).

Fv_0_ (N)	CI (N · s^−1^)	BD (s)	PD (s)	TD (s)	MBP (W · kg^−1^ · s^−1^)	MPP (W · kg^−1^ · s^−1^)	II (N · s^−1^)	iDSI (ratio)	fDSI (ratio)
Median	1788.04	221.65	0.30	0.27	0.58	7.24	28.45	97.68	0.69	0.68
IQR	729.40	70.28	0.06	0.02	0.05	2.74	6.68	45.57	0.23	0.17
10^th^ %	1345.68	139.67	0.374	0.308	0.635	5.66	24.98	183.90	0.54	0.56
20^th^ %	1435.2	165.86	0.362	0.295	0.622	5.80	26.24	207.20	0.55	0.60
30^th^ %	1543.40	189.38	0.325	0.283	0.607	6.07	27.73	246.41	0.57	0.63
40^th^ %	1692.76	203.33	0.321	0.278	0.588	6.44	28.30	277.79	0.60	0.67
50^th^ %	1867.87	222.74	0.304	0.272	0.580	7.55	28.59	316.58	0.69	0.71
60^th^ %	2062.89	228.67	0.306	0.267	0.576	8.30	29.56	352.00	0.73	0.79
70^th^ %	2106.43	239.88	0.287	0.257	0.566	8.39	32.94	375.26	0.78	0.80
80^th^ %	2176.56	250.80	0.281	0.255	0.557	8.79	34.99	400.29	0.86	0.83
90^th^ %	2218.53	265.34	0.268	0.249	0.550	9.50	37.75	419.00	1.03	0.87

IQR: Interquartile Range; F_V0_: Force at zero center-of-mass velocity (N); BD: Braking phase duration (s); PD: Propulsion phase duration (s);TD: Total duration (s); MBP: Mean braking phase power over time (W · kg^−1^ · s^−1^); MPP: Mean propulsion phase power over time (W · kg^−1^ · s^−1^); II: Impulse from IMTP (N · s^−1^); iDSI: impulse-derived dynamic strength indices; fDSI: force-derived dynamic strength indices. CI: CMJ propulsion phase Impulse(N · s^−1^).

### Statistical Analyses

All analyses were performed using R in RStudio (version 2024.04; RStudio PBC, Boston, MA, USA) [27]. Data normality was assessed using the Shapiro–Wilk test, implemented via the *stats::shapiro*. test function. As DSI values were non-normally distributed (*p* < 0.05), comparisons between fDSI and iDSI were conducted using the Wilcoxon signed-rank test via the *stats::wilcox*. test function. As a consequence of normality findings, results are reported as medians with interquartile ranges (IQR).

Agreement between training recommendations derived from fDSI and iDSI was evaluated using Cohen’s kappa, calculated with the *irr::kappa2* function. Interpretation thresholds were defined as poor (κ ≤ 0.40), moderate (0.41–0.60), substantial (0.61–0.80), and almost perfect (κ > 0.80) [28]. Measurement reliability was assessed using typical error (TE), coefficient of variation (CV), and intraclass correlation coefficients (ICC). ICCs were computed using the *irr::icc* function under a two-way random-effects, absolute-agreement, single-measures model [ICC (2,1)], and interpreted as poor (< 0.50), moderate (0.50–0.75), good (0.75–0.90), or excellent (> 0.90) [29]. Correlation analyses were performed using the stats::cor. test function, applying Pearson’s r for normally distributed variables and Spearman’s ρ otherwise. Correlation magnitudes were interpreted using Hopkins’ thresholds [30]: trivial (< 0.10), small (0.10–0.29), moderate (0.30–0.49), large (0.50–0.69), very large (0.70–0.89), and nearly perfect (≥ 0.90).

## RESULTS

All CMJ and IMTP performance variables demonstrated excellent within-session reliability. Intraclass correlation coefficients (ICCs) for CMJ ranged from 0.895 to 0.931 (95% CI: 0.832–0.987), while IMTP values ranged from 0.815 to 0.956 (95% CI: 0.783–0.990). For derived indices, ICCs were 0.733 (95% CI: 0.692–0.753) for iDSI and 0.712 (95% CI: 0.673–0.736) for fDSI.

The coefficient of variation (CV), calculated from typical error (TE), ranged from 3.5% to 8.2% (95% CI: 3.1%–8.7%) for CMJ and 2.7% to 7.5% (95% CI: 2.1%–8.3%) for IMTP. For the DSI metrics, CVs were 9.7% (95% CI: 9.1%–10.8%) for iDSI and 11.3% (95% CI: 9.8%–11.9%) for fDSI.

A Wilcoxon signed-rank test indicated a significant difference between iDSI and fDSI values (*Z* = –3.72, *p* < 0.001). Due to nonnormal data distributions, median and interquartile ranges were used for descriptive statistics. Full distributions of key performance metrics are provided in Table 1.

**FIG. 3:**
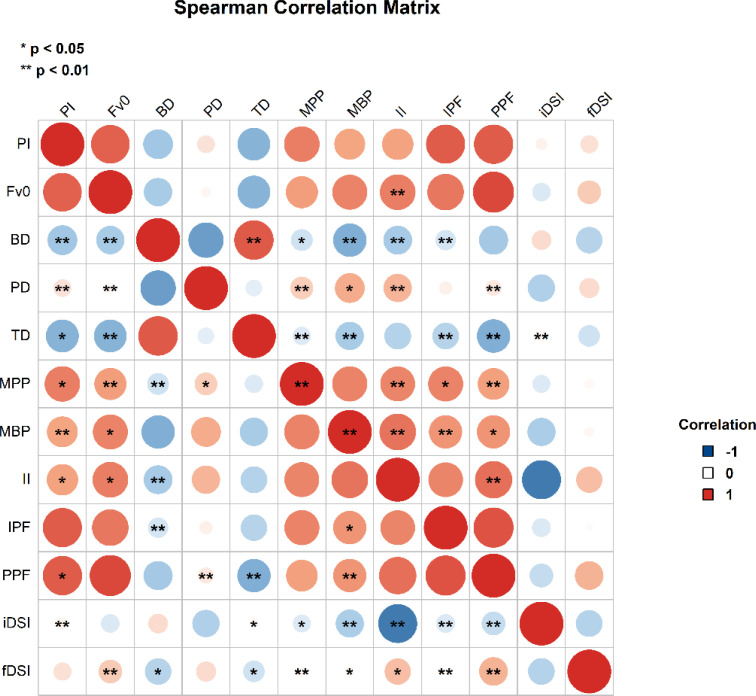
Heatmap illustrating the correlation between DSIs and selected CMJ performance variables. The variables are defined as follows: F_V0_: Force at zero center-of-mass velocity (N); BD: Braking phase duration (s); PD: Propulsion phase duration (s);TD: Total duration (s); MBP: Mean braking phase power over time (W · kg^−1^ · s^−1^); MPP: Mean propulsion phase power over time (W · kg^−1^ · s^−1^); II: Impulse from IMTP (N · s^−1^); IPF: IMTP peak force (N); PPF: CMJ peak force (N). iDSI: impulse-based dynamic strength index, and fDSI: force-based dynamic strength index; *indicates statistical significance. All correlations in the heat map were computed using Spearman’s ρ due to non-normal data distributions.

A correlation heatmap (Figure 3) was generated to evaluate the relationships among key performance variables. iDSI demonstrated weak correlations with CMJ performance metrics, with all *r*_s_ below 0.04. Similarly, fDSI exhibited weak or negligible associations with most variables (*r*_s_ < 0.04, *p* > 0.005), except for a moderate positive correlation with PPF (*r*_s_ = 0.42, *p* < 0.005). In contrast, F_V0_ showed strong, statistically significant correlations with several performance measures: PPF *(r*_s_ = 0.87, *p* < 0.005, 95% CI [0.70, 0.95]), CI (*r*_s_ = 0.77, *p* < 0.05, 95% CI [0.50, 0.90]), MPP (*r*_s_ = 0.53, *p* < 0.005, 95% CI [0.11, 0.79]), and MBP (*r*_s_ = 0.63, *p* < 0.005, 95% CI [0.26, 0.84]). Despite a moderate correlation between iDSI and fDSI (*r*_s_ = 0.47, *p* < 0.005, 95% CI [0.03, 0.76]), both indices showed limited associations with key force-time metrics (*rs* < 0.40). Moreover, a linear regression analysis examining the predictive relationship between iDSI and fDSI yielded a poor model fit (*β:* 0.243; 95% CI: −0.224 to 0.619, R^**2**^ = 0.059, *p* = 0.32), indicating substantial divergence between the constructs captured by these two indices.

**FIG. 4:**
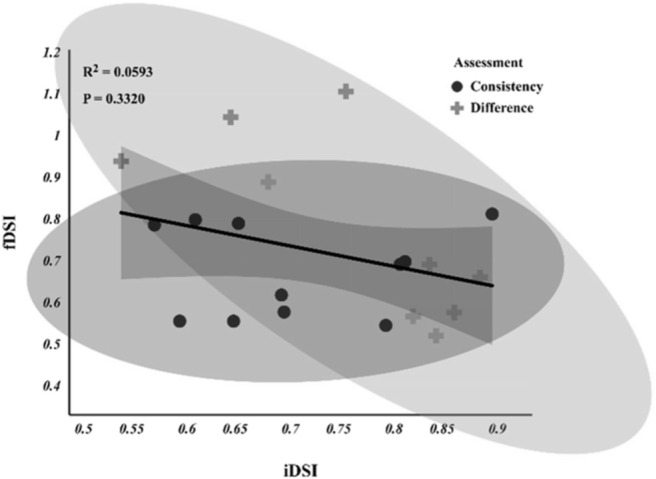
Scatterplot of dynamic strength indices (DSIs). The x-axis shows iDSI values, and the y-axis shows fDSI values. Shaded regions represent the 95% confidence interval bounds. Dots indicate athletes with consistent training recommendations (n = 11), while crosses denote conflicting recommendations (n = 9).

Agreement in training recommendations based on iDSI and fDSI were consistent for 11 athletes, but discrepancies were observed in 9 cases (Figure 4). The agreement between the two indices was moderate (*κ* = 0.52, 95% CI: [0.38, 0.66]). Although the kappa statistic showed moderate agreement between the two measures on training recommendations (*κ* = 0.52), there was still a difference in 45% of cases.

**FIG. 5:**
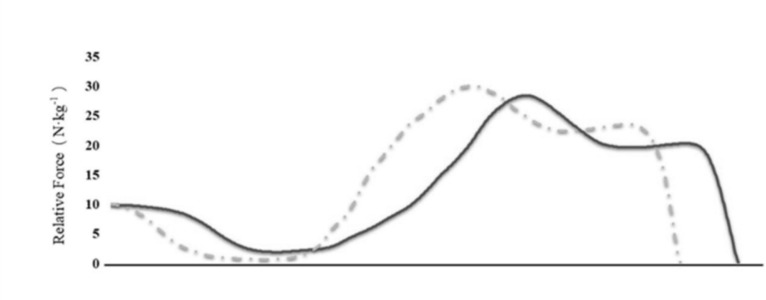
Force-time curves for Athlete X and Athlete Y. The solid line represents Athlete X, and the dashed line represents Athlete Y.

A case study analysis was conducted to explore individual differences in force-time characteristics. Comparison of two athletes revealed notable variability in percentile rankings across key performance metrics. Percentile ranks were used to standardize variables expressed in different units and to provide a dose-related representation of relative performance capacities, thereby facilitating interathlete comparisons in the case study analyses. Athlete X generally ranked near the 50^th^ percentile for most variables, except for F_V0_ and MBP, where deviations were observed (Table 2, Figure 5).

**TABLE 2:** The variables of the CMJ test for Athlete X and Athlete Y, along with their respective dynamic strength indexes (DSIs) and percentile ranges.

	Athlete X		Athlete Y	

	Magnitude	Percentile	Magnitude	Percentile
Height (cm)	185.3	—	172.5	—
Body Mass (kg)	77.53	—	68.55	—
F_V0_ (N)	2219.43	90^th^	2047.22	60^th^
CI (N · s^−1^)	221.56	50^th^	232.7	60^th^
BD (s)	0.304	50^th^	0.285	30^th^
PD (s)	0.275	50^th^	0.267	40^th^
TD (s)	0.579	40^th^	0.552	10^th^
MPP (W · kg^−1^ · s^−1^)	28.62	50^th^	37.87	90^th^
MBP (W · kg^−1^ · s^−1^)	8.72	80^th^	9.85	90^th^
iDSI	0.57	30^th^	.0.55	20^th^
fDSI	0.84	80^th^	0.58	10^th^

F_V0_: Force at zero center-of-mass velocity (N); BD: Braking phase duration (s); PD: Propulsion phase duration (s); TD: Total duration (s); MBP: Mean braking phase power over time (W · kg^−1^ · s^−1^); MPP: Mean propulsion phase power over time (W · kg^−1^ · s^−1^); IPF: IMTP peak force (N); CPF: CMJ peak force (N); CI: CMJ propulsion phase Impulse (N · s^−1^).

In the CMJ test, Athlete Y exhibited higher MPP (37.87 W · kg^−1^ · s^−1^) and MBP (9.85 W · kg^−1^ · s^−1^) compared to Athlete X (28.62 W · kg^−1^ · s^−1^ and 8.72 W · kg^−1^ · s^−1^, respectively). Force-time curve visualization showed that although both athletes demonstrated similar slopes during the Unweighting Phase, Athlete Y exhibited a visibly steeper forcetime slope during the braking phase. Athlete Y also generated greater braking and propulsive phase forces than Athlete X. Based on DSI scores, Athlete X had an fDSI ratio of 0.84 and an iDSI ratio of 0.57.

## DISCUSSION

This study examined force-time characteristics to evaluate iDSI and fDSI in relation to athletic performance. It assessed consistency in training recommendations and explored individualized applications via case studies. Although this study was conducted with sliding sports athletes, the primary determinant of performance in this discipline “start acceleration” is biomechanically similar to the linear explosive strength required in sprinting. Therefore, while the direct applicability of our findings is most relevant to sliding sports, the mechanistic insights may also provide a reference value for other sports that depend on rapid force production and linear acceleration. Findings showed weak correlations between DSI types and CMJ metrics, highlighting limitations in neuromuscular assessment validity. A 45% discrepancy in training prescriptions was noted between the two indices. Case studies emphasized the value of individualized force-time profiling and using multiple variables over relying solely on DSI.

Previous research [31] has demonstrated that DSIs computed over specific time intervals offer superior insights into lower-limb muscle function and training prescription. However, the current study [32, 33] suggests that both iDSI and fDSI have inherent validity limitations in assessing neuromuscular function (*Z* = -3.72, *p* < 0.01). Notably, at the zero-velocity transition point, generated force exhibited strong associations with multiple force-time performance metrics [34]. The weak correlation between PD and CI suggests that different jump strategies influence force-time curve characteristics (as demonstrated in the case study) [35, 36]. Both DSI indices showed weak correlations with selected force-time performance metrics. One possible explanation for the weak association observed in this study is the reliance on static force variables derived from the IMTP test. Specifically, no strong correlations were found between IMTP variables and dynamic performance measures such as CMJ propulsion force, with a substantial proportion of variance remaining unexplained. This highlights the limited capacity of IMTP-derived metrics to predict dynamic outputs. These findings are in line with those of Rago et al. [37], who similarly reported a lack of association between IMTP characteristics and dynamic outputs. However, this is a point of contention, as a number of other studies have shown significant relationships between IMTP-derived metrics and various dynamic performance outcomes, including sprint speed, jump height, and change of direction ability [37]. This inconsistency in the literature suggests that the strength of the relationship between isometric and dynamic performance may be influenced by several contextual factors, such as the characteristics of the test population, the specificity of the movement tasks, or the particular variables selected for analysis. Furthermore, a key factor is that DSIs are ratio data, which combine CMJ and IMTP variables, and consequently, is likely to result in larger measurement error (as our findings illustrate) and has been shown in previous DSI studies also [38]. Therefore, it is recommended to focus on the individual components rather than solely the ratio itself, as this enables a deeper understanding of physical profiles or any training effects [39]. DSIs provide only a general measure of strength expression and do not sufficiently reflect the timing or quality of jump phases such as braking and propulsion. Moreover, maximal force expression is inherently multi-facetted and cannot be fully captured by combining only two tests. Therefore, DSIs should be applied as a supplementary reference rather than the exclusive determinant for training program design.

The discrepancies in training recommendations generated by iDSI and fDSI were further examined through scatterplot visualization. Among the 20 athletes assessed, 11 exhibited agreements between iDSI and fDSI-based prescriptions, while 9 displayed conflicting recommendations. Typically, DSI values greater than 0.80 indicate a need for peak strength training, whereas values below 0.60 suggest a focus on dynamic force expression. However, the moderate agreement between iDSI and fDSI (*κ* = 0.52, 95% CI [0.38, 0.66]) suggests that relying solely on DSI for training prescription may be problematic. While the DSI may indicate a need for strength-focused training, its direct application must be contextually adapted throughout the training year. For instance, during congested competition phases, it may still be appropriate to follow the DSI-informed training direction. Rather than discarding DSI-based recommendations during congested competition periods, these measures should be embedded within a flexible, periodized framework that considers athlete recovery, competition load, and technical–tactical priorities. While force–time derived indices and detailed force–time characteristics are resource-intensive and time-consuming to assess, they offer substantial scientific value. As such, their optimal application may be as periodic or off-season evaluations to inform long-term training strategies, complemented by simpler and more accessible metrics for routine in-season monitoring. Given these discrepancies, a case study analysis was conducted to explore the practical implications of individualized force-time assessments. Athlete Y demonstrated superior braking and propulsion force characteristics, as reflected by higher braking force and greater propulsion impulse. In contrast, Athlete X employed a more gradual braking strategy, characterized by lower peak braking phase force and a less pronounced transition into the propulsion phase. These findings challenge conventional interpretations of DSI scores. Sheppard et al. [26] suggested that athletes with fDSI > 0.80 should prioritize maximal strength training, noting that ballistic training is itself a form of strength training and that both maximal strength–oriented and ballistic approaches can contribute to improvements in force production. Maximal strength and ballistic-focused methods should not be seen as mutually exclusive, but their relative emphasis should vary according to the athlete’s neuromuscular profile. For example, in this study, Athlete X, despite a relatively high fDSI, appeared to benefit more from ballistic training, indicating that higher fDSI values do not always reflect steeper force–time curves. In contrast, Athlete Y, whose force–time profile closely matched the theoretical optimum, would likely benefit from a hybrid program combining maximal strength and ballistic training. These cases underscore the need for individualized prescriptions that extend beyond DSI values alone.

In practice, high-performance teams can address this by adopting an integrated framework that applies multiple performance indicators to classify and evaluate athlete profiles. Using a range of complementary measures enables more accurate diagnosis of underlying strength qualities and supports the design of tailored prescriptions. In team settings, these assessments can be further contextualized with percentile-based rankings and dynamic visualization tools, allowing practitioners to benchmark athletes, monitor progress, and adapt programs in an athlete-centered manner.

These findings highlight the limited standalone applicability of DSI values in practice and underscore the need to interpret them within a broader neuromechanical context, complemented by additional force–time variables. As ratio metrics, iDSI and fDSI inherently carry limitations, including error propagation from their constituent measures and sensitivity to small fluctuations in either numerator or denominator. Future research may enhance the reliability of these indices through methodological refinements, such as stricter standardization of testing protocols, averaging across multiple trials, and the use of phase-specific impulse ratios. Practitioners should integrate individualized metrics—such as percentile-based DSI rankings, normalized force-time profiles, and neuromuscular characteristics—when planning training. The application of fDSI and iDSI across sports requires careful consideration of sport-specific biomechanical and physiological demands. From an applied perspective, the integration of fDSI and iDSI with phase-specific force-time variables provides practitioners with actionable insights. For instance, an athlete exhibiting low DSI but high IMTP impulse may benefit from ballistic or plyometric interventions to improve force transfer. Conversely, those with high DSI but limited isometric force capacity may require strength-focused interventions. Such individualized profiling aligns with contemporary best practices in athlete monitoring and training prescription. By contextualizing these indices, performance staff can make more informed decisions on load management and return-to-play criteria, ensuring athlete-specific interventions are both targeted and effective.

## CONCLUSIONS

Practitioners should avoid interpreting DSI values in isolation, as changes in CMJ or IMTP can distort the ratio and misguided training decisions. A better approach is to examine underlying force-time metrics to identify specific neuromuscular strengths and weaknesses. Sport context matters: explosive force is critical in basketball, football, and sprinting, while maximal force is more relevant in throwing events. Therefore, applying fDSI or iDSI requires sport-specific interpretation. Incorporating additional markers—such as stretchshortening cycle efficiency and time-normalized force profiles—supports a more individualized, performance-relevant model.

## Data Availability

The datasets generated and analyzed during the current study are not publicly available but are available from the corresponding author on reasonable request.
